# Secular trends of population attributable risk of overweight and obesity for hypertension among Chinese adults from 1991 to 2011

**DOI:** 10.1038/s41598-021-85794-2

**Published:** 2021-03-18

**Authors:** Jian Gou, Huiying Wu

**Affiliations:** Department of Nursing, Shengjing Hospital of China Medical University, Shenyang, 110004 Liaoning Province People’s Republic of China

**Keywords:** Epidemiology, Cardiology

## Abstract

We determined if the increasing trend in hypertension can be partly attributed to increasing prevalence of overweight/obesity in China over the past two decades. Data were collected from 1991 to 2011 and the population attributable risk (PAR), which is used to estimate the intervention effect on hypertension if overweight/obese, were eliminated. Linear regression was used to evaluate the secular trends. The age-standardized prevalence of overweight and obesity increased by 26.32% with an overall slope of 1.27% (95% CI: 1.12–1.43%) per year. Hypertension also increased by 12.37% with an overall slope of 0.65% (95% CI: 0.51–0.79%) per year. The adjusted ORs of overweight/obesity for hypertension across the survey years remained unchanged; however, the trend in PAR increased steadily from 27.1 to 44.6% with an overall slope of 0.81% (95% CI: 0.34–1.28%) per year (*P* = 0.006). There was no significant gender difference in the slopes of increasing PAR, as measured by regression coefficients (β = 0.95% vs. β = 0.63% per year, *P* = 0.36). Over the past two decades, the increase in the prevalence of hypertension in China was partly attributed to the overweight/obesity epidemic, which highlights the importance of controlling weight and further reducing the burden of hypertension.

## Introduction

Hypertension is a well-known risk factor for cardiovascular disease (CVD), which is the leading cause of death in China^1^. According to the 2017 China Cardiovascular Disease Report, there are currently 290 million CVD patients and 270 million adult hypertensive patients^2^. One recent study indicated that the prevalence of hypertension has reached 44.1% (range, 43.9–44.2%) among Chinese adults ≥ 35 years of age^3^. Thus, reducing the burden of disease associated with hypertension has been identified as a public health priority in China, as well as worldwide^4^. Despite the higher prevalence of hypertension, understanding the epidemic status of determinants of hypertension will facilitate a reduction in incident hypertension, which is also an urgent issue.

Overweight and obesity together constitute a global public health challenge^5^. In addition to the increased risk of overall mortality, overweight and obesity are associated with an increased risk for multiple morbidities, and the risk of developing hypertension increases as the body mass index (BMI) increases^6^. A number of studies, however, have demonstrated that trends in hypertension among adults are not necessarily accompanied by the increase in excess body weight over the past two decades^7–10^. Nagai et al.^7^ reported that the age-standardized prevalence of overweight and obesity increased 17.0% from 1980 to 2010, along with a 4.1% decrease in hypertension^7^. In addition, a recent study involving 943,128 Chinese children between the 7 and 17 years of age showed dramatic increases in the prevalence of overweight children from 1995 to 2014, the prevalence of hypertension remained relatively stable. Of note, the population attributable risk (PAR) of hypertension due to being overweight steadily increased from 6.3% in 1995 to 19.2% in 2014. PAR indicates the benefit from a public health perspective because the PAR aids in prioritizing health budgets and distribution of resources depending on the proportion of outcome attributed to a particular exposure^11^. The PAR reflects what percent of the disease incidence in the entire population will be prevented if a specific risk factor is eliminated. From a public health perspective, the PAR is often both a critical issue and the question that is raised by policymakers and those responsible for funding prevention programs. The PAR reflects how a program will change the disease burden on the healthcare system or the burden of suffering within the entire community and not just in exposed individuals.

The secular trend in the PAR of hypertension due to being overweight and obese in Chinese adults has not been established. The secular trends in overweight and hypertension data in the past two decades are limited.

Therefore, we used the data from the China Health and Nutrition Survey (CHNS) to analyze the secular trends in the prevalence of hypertension and overweight/obesity, and the PAR of overweight and obesity for hypertension from 1991 to 2011. We hypothesized that the increasing trend in hypertension over the past two decades could be partly attributed to increasing prevalence of overweight and obesity in China.

## Methods

### Study participants

The CHNS is an ongoing open cohort study of the National Institute for Nutrition and Health (Chinese Center for Disease Control and Prevention) in collaboration with the Population Center of the University of North Carolina in the United States.

The survey covers nine provinces that vary substantially in geography, economic development, public resources, and health indicators. A multistage, random cluster process was used to draw the samples surveyed in each of the provinces. Counties in the nine provinces were stratified by income (low, middle, and high) and a weighted sampling scheme was used to randomly select four counties in each province. In addition, the provincial capital and a lower income city were selected when feasible. In two provinces, other large cities had to be selected. Villages and townships within the counties and urban and suburban neighborhoods within the cities were selected randomly. Between 1989 and 1993 there were 190 primary sampling units, and a new province and its sampling units were added in 1997. There were approximately 4400 households in the overall survey involving approximately 19,000 individuals.

The first round of the CHNS, including household, community, and health/family planning facility data, was collected in 1989. Six additional panels were collected in 1991, 1993, 1997, 2000, 2004, and 2006. Since the 1993 survey, all new households formed from sample households were added. Since 1997, new households in the original communities were also added to replace households no longer participating in the study. Since 1997, new communities in original provinces have also been added to replace sites no longer participating. A new province was added in 1997 when one province was unable to participate. The dropped province returned to the study in 2000.

In 1989, the CHNS surveyed 3795 households and 15,907 individuals. Health and nutritional data were collected from preschoolers and adults 20–45 years of age. The 1991 CHNS surveyed individuals belonging to the original sample households, which resulted in a total of 3619 households and 14,778 individuals. Since 1993, all new households formed from sample households that were located in sample areas were added to this sample. Since the 1997 CHNS, all newly-formed households that were located in sample areas and additional households to replace the households no longer participating were added to the sample. New communities were also added to replace communities no longer participating, and Heilongjiang province was added. Since the 2011 CHNS, 3 megacities (Beijing, Chongqing, and Shanghai) were added. The survey was approved by the Institutional Review Committees of the University of North Carolina at Chapel Hill and the National Institute for Nutrition and Health (Chinese Center for Disease Control and Prevention).

**Figure 1:**
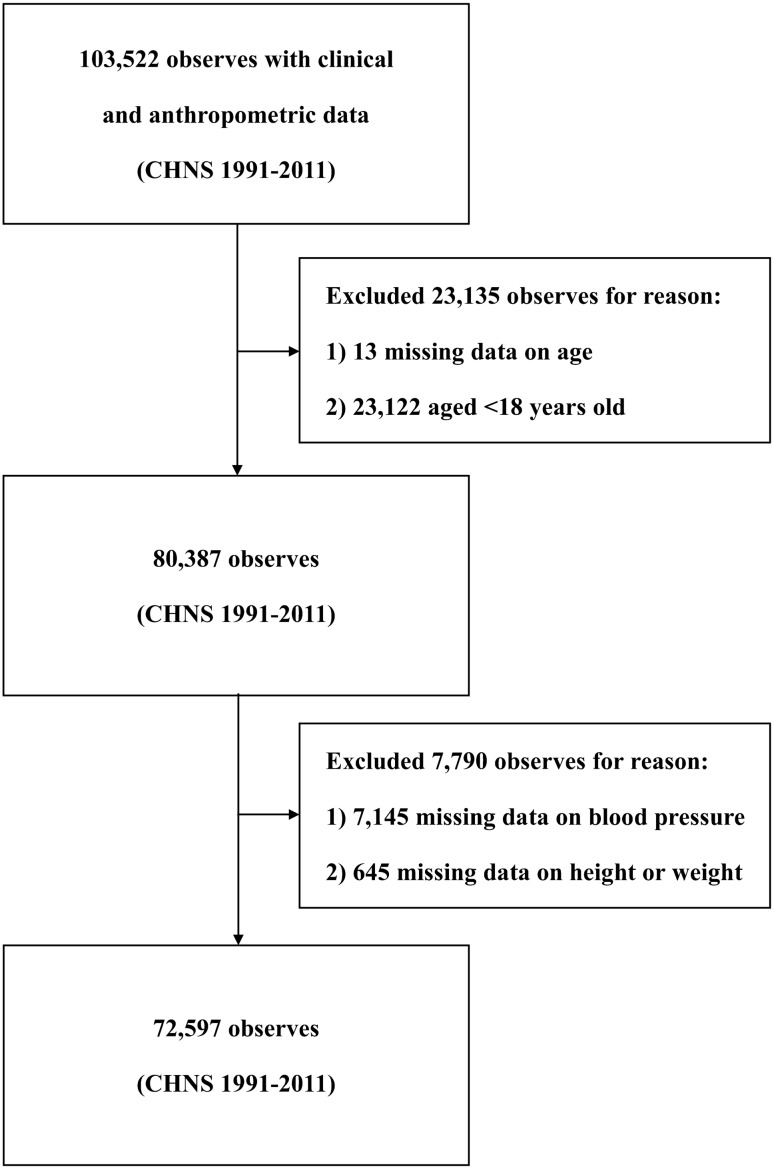
Flow chart illustrating the sample selection for the present study.

The sample selection process for the present study is summarized in Fig. 1. Participants < 18 years of age or participants with missing key variables were excluded. Basic demographic information included age (years) and gender. Physical measures included height (m), weight (kg), and blood pressure (BP in mmHg). Other data, such as current smoking and alcohol consumption were also collected in these surveys.

All methods were carried out in accordance with relevant guidelines and regulations. Informed consent was obtained from all subjects.

### BMI

Weight and height were measured without hats, shoes, and heavy clothes. The BMI was calculated using the following formula: BMI = weight in kg/(height in m)^2^. The BMI was categorized into 3 levels based on Chinese standards: < 18.5 kg/m^2^; 18.5–23.9 kg/m^2^; and ≥ 24 kg/m^2^, which corresponded to underweight, normal, and overweight and obesity, respectively^12^.

### BP measurement

BP was measured by trained examiners using a mercury sphygmomanometer with a suitable cuff size according to a standard protocol. The BP of the right arm was thrice-measured in the sitting position after a ≥ 10-min rest. Hypertension was defined as a mean systolic BP (SBP) and/or diastolic BP (DBP) ≥ 140/90 mmHg or current treatment with antihypertensive medications.

### Statistical analysis

Descriptive statistics were calculated for all variables across all survey years. The age-standardized prevalence of overweight and obese, and hypertension among men and women were calculated by the direct method using data from the 2000 World Census with weights of 0.143, 0.166, 0.168, 0.121, 0.080, and 0.100 for 6 age groups (18–29, 30‐39, 40‐49, 50–59, 60–69, and ≥ 70 years, respectively)^13^. Binary logistic regression was used to calculate the odds ratios (ORs) and 95% confidence intervals (CIs) of overweight and obese in hypertensive participants, with the normal weight participants as the reference group across all survey years. Age, current smoking and alcohol consumption were fully adjusted in these multiple logistic regression models. PAR was calculated as follows: PAR = {Prevalence × (OR-1)]/[Prevalence × (OR-1) + 1}^14^, which implies a theoretical causal relationship between overweight/obesity and hypertension^15^. We also used the PAR to estimate intervention effects on hypertension if overweight and obesity were eliminated in adults^11^. The linear regression was used to evaluate the secular trends of prevalence, OR, and PAR over years. The slope across survey years was used as a surrogate indicator of the average increased velocity, and we detected significant increasing trends across the eight survey years. The difference in slopes was examined using t-tests, as recommended by Kleinbaum^16^. All statistical analyses were performed with SAS (version 9.4; SAS Institute Inc., Cary, NC, USA) and SPSS statistical software (version 22.0; IBM Corp., Armonk, NY, USA). A *P* value < 0.05 indicated statistical significance.

## Results

The characteristics of the study participants for each survey are shown in Table 1. Increasing trends in mean age and proportions of antihypertensive drug use were observed in both genders. An increasing trend in the mean BMI and a decreasing trend in the proportion of thinness were also observed. There were clear increases in SBP and DBP levels from 1991 to 2011.

**Table 1:** Baseline characteristics of study participants.

	1991	1993	1997	2000	2004	2006	2009	2011	*P* value
**Total**									
Number of participants	8382	7841	8461	9462	8749	8854	8358	12,490	
Age (years)	40.83 ± 15.72	41.67 ± 15.66	43.28 ± 15.80	44.80 ± 15.47	47.91 ± 15.36	49.15 ± 15.18	50.46 ± 15.58	51.01 ± 15.21	< 0.001
Weight (kg)	55.27 ± 9.25	55.98 ± 9.23	57.34 ± 10.11	59.03 ± 10.55	59.73 ± 10.98	60.17 ± 10.99	60.60 ± 11.36	62.45 ± 12.88	< 0.001
Height (cm)	159.51 ± 8.35	159.78 ± 8.26	160.12 ± 8.35	160.68 ± 8.41	160.64 ± 8.64	160.83 ± 8.64	160.88 ± 8.66	161.37 ± 9.15	< 0.001
BMI (kg/m^2^)	21.67 ± 2.86	21.87 ± 2.85	22.30 ± 3.12	22.80 ± 3.25	23.07 ± 3.39	23.19 ± 3.56	23.33 ± 3.49	23.93 ± 4.61	< 0.001
**BMI categories (kg/m**^**2**^**), n (%)**									
< 18.5	868 (10.4)	686 (8.7)	683 (8.1)	643 (6.8)	556 (6.4)	542 (6.1)	556 (6.7)	626 (5.0)	< 0.001
18.5–23.9	5964 (71.2)	5532 (70.6)	5580 (65.9)	5777 (61.1)	5048 (57.7)	5001 (56.5)	4495 (53.8)	6291 (50.4)	
≥ 24	1550 (18.5)	1623 (20.7)	2198 (26.0)	3042 (32.1)	3145 (35.9)	3311 (37.4)	3307 (39.6)	5573 (44.6)	
SBP (mmHg)	114.90 ± 18.55	115.55 ± 17.68	118.79 ± 18.02	119.76 ± 17.94	122.26 ± 18.69	121.70 ± 18.03	124.65 ± 18.88	124.53 ± 17.74	< 0.001
DBP (mmHg)	74.40 ± 11.40	75.62 ± 11.16	77.11 ± 11.01	77.67 ± 11.10	78.74 ± 11.28	78.90 ± 11.01	80.15 ± 11.13	79.30 ± 10.69	< 0.001
Antihypertensive medications, n (%)	196 (2.3)	207 (2.6)	235 (2.8)	450 (4.8)	581 (6.6)	689 (7.8)	889 (10.6)	1660 (13.3)	< 0.001
Smoking, n (%)	2735 (32.6)	2480 (31.6)	2599 (30.7)	2798 (29.6)	2505 (28.6)	2385 (26.9)	2327 (27.8)	3261 (26.1)	< 0.001
Drinking, n (%)	3157 (37.7)	2780 (35.5)	3022 (35.7)	3227 (34.1)	2872 (32.8)	2815 (31.8)	2761 (33.0)	4215 (33.7)	< 0.001
**Men**									
Number of participants	3968	3717	4091	4512	4153	4155	3946	5828	
Age (years)	40.80 ± 15.54	41.59 ± 15.54	42.84 ± 15.78	44.54 ± 15.52	47.58 ± 15.38	48.87 ± 15.18	50.33 ± 15.50	51.18 ± 15.10	< 0.001
Weight (kg)	58.77 ± 8.87	59.61 ± 8.85	61.06 ± 9.91	62.99 ± 10.49	63.91 ± 10.86	64.47 ± 10.95	65.03 ± 11.35	67.38 ± 13.15	< 0.001
Height (cm)	165.35 ± 6.39	165.58 ± 6.30	165.88 ± 6.44	166.48 ± 6.48	166.56 ± 6.66	166.74 ± 6.69	166.80 ± 6.71	167.41 ± 7.58	< 0.001
BMI (kg/m^2^)	21.45 ± 2.64	21.70 ± 2.65	22.13 ± 2.97	22.66 ± 3.14	22.97 ± 3.22	23.12 ± 3.25	23.30 ± 3.39	24.01 ± 4.73	< 0.001
**BMI categories (kg/m**^**2**^**), n (%)**									
< 18.5	408 (10.3)	317 (8.5)	329 (8.0)	287 (6.4)	244 (5.9)	246 (5.9)	252 (6.4)	256 (4.4)	< 0.001
18.5–23.9	2970 (74.8)	2753 (74.1)	2812 (68.7)	2860 (63.4)	2458 (59.2)	2372 (57.1)	2111 (53.5)	2893 (49.6)	
≥ 24	590 (14.9)	647 (17.4)	950 (23.2)	1365 (30.3)	1451 (34.9)	1537 (37.0)	1583 (40.1)	2679 (46.0)	
SBP (mmHg)	117.13 ± 17.62	117.59 ± 16.40	120.33 ± 16.53	121.49 ± 16.56	124.02 ± 17.32	123.46 ± 16.82	125.91 ± 17.47	126.24 ± 16.35	< 0.001
DBP (mmHg)	75.95 ± 11.18	77.12 ± 10.73	78.43 ± 10.68	79.14 ± 10.65	80.21 ± 10.86	80.46 ± 10.77	81.79 ± 10.98	80.89 ± 10.46	< 0.001
Antihypertensive medications, n (%)	77 (1.9)	103 (2.8)	111 (2.7)	183 (4.1)	247 (5.9)	300 (7.2)	389 (9.9)	736 (12.6)	< 0.001
Smoking, n (%)	2549 (64.2)	2301 (61.9)	2404 (58.8)	2581 (57.2)	2318 (55.8)	2219 (53.4)	2211 (56.0)	3067 (52.6)	< 0.001
Drinking, n (%)	2571 (64.8)	2301 (61.9)	2568 (62.8)	2739 (60.7)	2472 (59.5)	2428 (58.4)	2365 (59.9)	3455 (59.3)	< 0.001
**Women**									
Number of participants	4414	4124	4370	4950	4596	4699	4412	6662	
Age (years)	40.86 ± 15.89	41.74 ± 15.77	43.70 ± 15.81	45.05 ± 15.42	48.20 ± 15.35	49.40 ± 15.18	50.58 ± 15.64	50.87 ± 15.30	< 0.001
Weight (kg)	52.13 ± 8.41	52.70 ± 8.29	53.86 ± 9.00	55.43 ± 9.24	55.96 ± 9.65	56.36 ± 9.52	56.64 ± 9.80	58.13 ± 10.94	< 0.001
Height (cm)	154.27 ± 6.12	154.56 ± 6.02	154.72 ± 6.01	155.39 ± 6.18	155.29 ± 6.47	155.61 ± 6.55	155.59 ± 6.51	156.09 ± 6.85	< 0.001
BMI (kg/m^2^)	21.86 ± 3.03	22.03 ± 3.02	22.45 ± 3.25	22.92 ± 3.34	23.16 ± 3.53	23.26 ± 3.82	23.36 ± 3.58	23.86 ± 4.51	< 0.001
**BMI categories (kg/m**^**2**^**), n (%)**									
< 18.5	460 (10.4)	369 (8.9)	354 (8.1)	356 (7.2)	312 (6.8)	296 (6.3)	304 (6.9)	370 (5.6)	< 0.001
18.5–23.9	2994 (67.8)	2779 (67.4)	2768 (63.3)	2917 (58.9)	2590 (56.4)	2629 (55.9)	2384 (54.0)	3398 (51.0)	
≥ 24	960 (21.7)	976 (23.7)	1248 (28.6)	1677 (33.9)	1694 (36.9)	1774 (37.8)	1724 (39.1)	2894 (43.4)	
SBP (mmHg)	112.90 ± 19.12	113.71 ± 18.57	117.34 ± 19.19	118.18 ± 18.98	120.67 ± 19.72	120.15 ± 18.90	123.52 ± 20.00	123.03 ± 18.75	< 0.001
DBP (mmHg)	73.02 ± 11.42	74.27 ± 11.37	75.88 ± 11.17	76.33 ± 11.33	77.41 ± 11.48	77.51 ± 11.04	78.69 ± 11.07	77.90 ± 10.70	< 0.001
Antihypertensive medications, n (%)	119 (2.7)	104 (2.5)	124 (2.8)	267 (5.4)	334 (7.3)	389 (8.3)	500 (11.3)	924 (13.9)	< 0.001
Smoking, n (%)	186 (4.2)	179 (4.3)	195 (4.5)	217 (4.4)	187 (4.1)	166 (3.5)	116 (2.6)	194 (2.9)	< 0.001
Drinking, n (%)	586 (13.3)	479 (11.6)	454 (10.4)	488 (9.9)	400 (8.7)	387 (8.2)	396 (9.0)	760 (11.4)	< 0.001

*BMI* Body mass index, *SBP* Systolic blood pressure, *DBP* Diastolic blood pressure.

**Figure 2:**
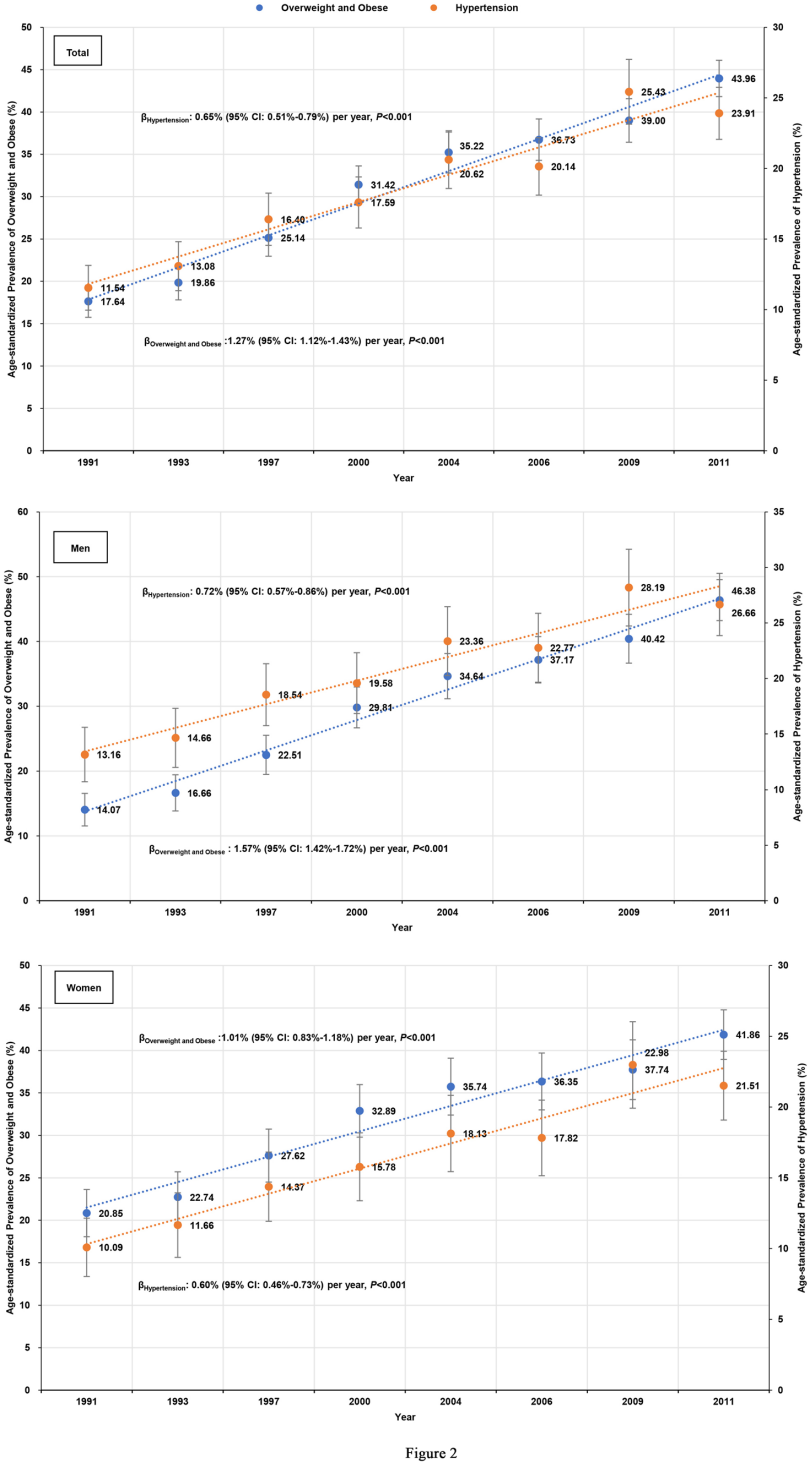
Secular trends in age-standardized prevalence of hypertension (95% CIs) and age-standardized prevalence of overweight/obesity.

The age-standardized prevalence of hypertension and overweight/obesity in each survey among men and women are shown in Fig. 2. The age-standardized prevalence of overweight/obesity increased during the past two decades, and hypertension increased in both genders. The age-standardized prevalence of overweight/obesity increased by a total of 26.32% (1991: 17.64%; 2011: 43.96%), 32.31% (1991: 14.07%; 2011: 46.38%) in men, and 21.01% (1991: 20.85%; 2011: 41.86%) in women. The prevalence of overweight/obesity increased significantly, with overall regression coefficients (β) of 1.27% (95% CI: 1.12–1.43%) per year. The slopes of increasing age-standardized prevalence of overweight/obesity, as measured by regression coefficients (β), were significantly greater in men than women (β = 1.57% per year vs. β = 1.01% per year, *P* < 0.001). The age-standardized prevalence of hypertension also increased by a total of 12.37% (1991: 11.54%; 2011: 23.91%), 13.50% (1991: 13.16%; 2011: 26.66%) in men, and 11.42% (1991: 10.09%; 2011: 21.51%) in women. The prevalence of hypertension increased with overall regression coefficients (β) of 0.65% (95% CI: 0.51–0.79%) per year. There was no significant difference in the slopes of increasing prevalence of age-standardized hypertension, as measured by regression coefficients (β), between men and women (β = 0.72% per year vs. β = 0.60% per year, *P* = 0.33).

**Figure 3:**
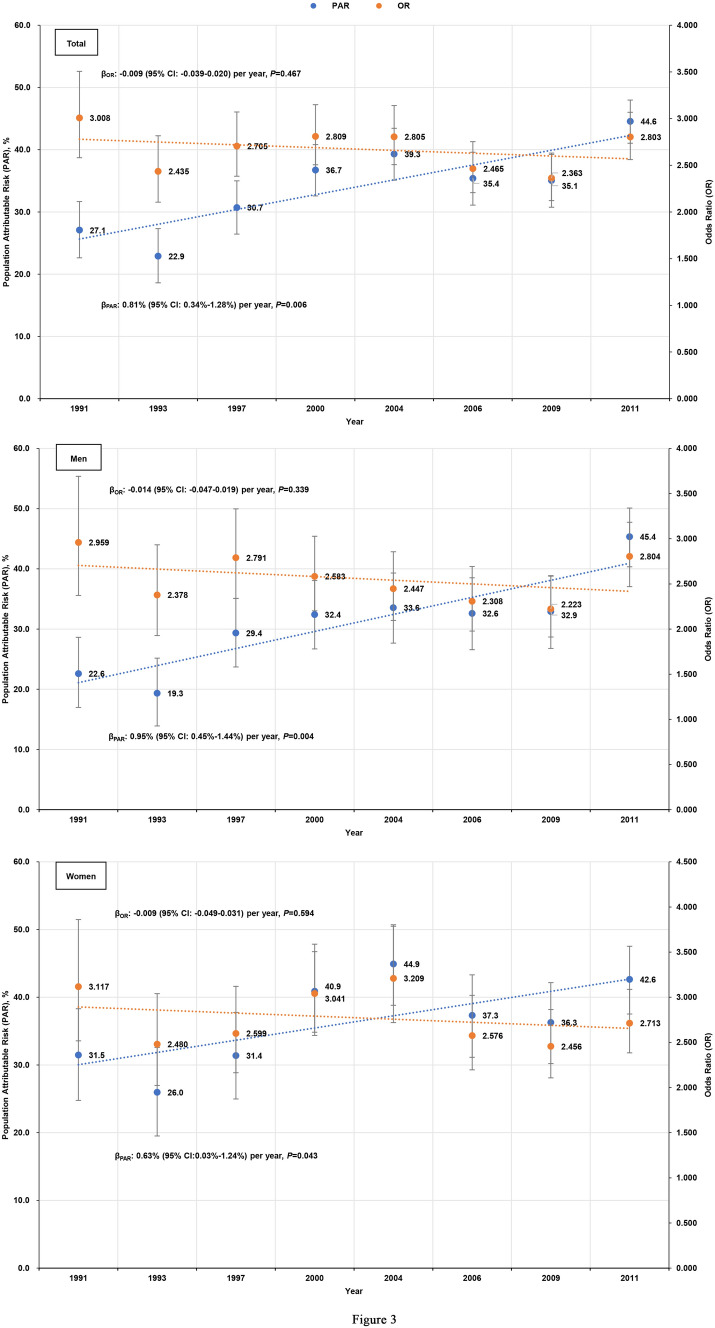
Secular trends in ORs (95% CIs) for hypertension in overweight/obese participants compared with normal weight participants. The ORs were adjusted for age, current smoking, and current alcohol consumption. Secular trends in PAR (95% CIs) of hypertension because of overweight/obesity.

The multivariable-adjusted ORs (95% CIs) for hypertension in overweight/obese participants compared with normal weight participants are shown in Table 2. There was a significant impact of overweight/obesity on hypertension in each survey; however, as shown in Fig. 3, the adjusted ORs and 95% CIs for the association between overweight/obesity and hypertension across survey years remained stable, with an overall slope of − 0.009 (95% CI: − 0.039–0.020) per year (*P* = 0.467). A similar trend was noted in men and women (β =  − 0.014 per year vs. β =  − 0.009 per year, *P* = 0.99). In 1991 and 2011, the multivariable-adjusted ORs (95% CIs) were a total of 3.008 (2.581–3.506) and 2.803 (2.561–3.068), 2.959 (2.372–3.692) and 2.804 (2.470–3.183) for men, and 3.117 (2.517–3.861) and 2.713 (2.384–3.087) for women, respectively. The trend in hypertension attributed to an increasing prevalence of overweight/obesity increased steadily from 27.1% to 44.6%, with an overall slope of 0.81% (95% CI: 0.34–1.28%) per year (*P* = 0.006). There was no significantly gender difference in slopes of increasing PAR, as measured by regression coefficients (β) (β = 0.95% per year vs. β = 0.63% per year, *P* = 0.36).

**Table 2:** Associations of overweight and obese with prevalent hypertension.

BMI (kg/ m^2^)	Total				Men				Women			
	Number, n (%)	β	OR (95%CI)	*P*	Number, n (%)	β	OR (95%CI)	*P*	Number, n (%)	β	OR (95%CI)	*P*
**1991**												
18.5–24	5964 (71.2)		1.000 (ref.)		2970 (74.8)		1.000 (ref.)		2994 (67.8)		1.000 (ref.)	
< 18.5	868 (10.4)	− 0.528	0.590 (0.459,0.758)	< 0.001	408 (10.3)	− 0.697	0.498 (0.349,0.712)	< 0.001	460 (10.4)	− 0.344	0.709 (0.497,1.011)	0.057
≥ 24	1550 (18.5)	1.101	3.008 (2.581,3.506)	< 0.001	590 (14.9)	1.085	2.959 (2.372,3.692)	< 0.001	960 (21.7)	1.137	3.117 (2.517,3.861)	< 0.001
**1993**												
18.5–24	5532 (70.6)		1.000 (ref.)		2753 (74.1)		1.000 (ref.)		2779 (67.4)		1.000 (ref.)	
< 18.5	686 (8.7)	− 0.412	0.662 (0.508,0.864)	0.002	317 (8.5)	− 0.303	0.739 (0.515,1.059)	0.099	369 (8.9)	− 0.532	0.587 (0.396,0.871)	0.008
≥ 24	1623 (20.7)	0.890	2.435 (2.105,2.816)	< 0.001	647 (17.4)	0.866	2.378 (1.928,2.933)	< 0.001	976 (23.7)	0.908	2.480 (2.023,3.040)	< 0.001
**1997**												
18.5–24	5580 (65.9)		1.000 (ref.)		2812 (68.7)		1.000 (ref.)		2768 (63.3)		1.000 (ref.)	
< 18.5	683 (8.1)	− 0.574	0.563 (0.437,0.725)	< 0.001	329 (8.0)	− 0.350	0.705 (0.501,0.991)	0.044	354 (8.1)	− 0.880	0.415 (0.284,0.605)	< 0.001
≥ 24	2198 (26.0)	0.995	2.705 (2.383,3.071)	< 0.001	950 (23.2)	1.026	2.791 (2.339,3.331)	< 0.001	1248 (28.6)	0.955	2.599 (2.164,3.121)	< 0.001
**2000**												
18.5–24	5777 (61.1)		1.000 (ref.)		2860 (63.4)		1.000 (ref.)		2917 (58.9)		1.000 (ref.)	
< 18.5	643 (6.8)	− 0.568	0.567 (0.435,0.739)	< 0.001	287 (6.4)	− 0.547	0.579 (0.398,0.841)	0.004	356 (7.2)	− 0.593	0.552 (0.379,0.806)	0.002
≥ 24	3042 (32.1)	1.033	2.809 (2.505,3.149)	< 0.001	1365 (30.3)	0.949	2.583 (2.202,3.028)	< 0.001	1677 (33.9)	1.112	3.041 (2.577,3.588)	< 0.001
**2004**												
18.5–24	5048 (57.7)		1.000 (ref.)		2458 (59.2)		1.000 (ref.)		2590 (56.4)		1.000 (ref.)	
< 18.5	556 (6.4)	− 0.166	0.847 (0.662,1.083)	0.185	244 (5.9)	− 0.292	0.747 (0.522,1.069)	0.111	312 (6.8)	− 0.084	0.920 (0.654,1.293)	0.631
≥ 24	3145 (35.9)	1.032	2.805 (2.507,3.140)	< 0.001	1451 (34.9)	0.895	2.447 (2.096,2.856)	< 0.001	1694 (36.9)	1.166	3.209 (2.719,3.787)	< 0.001
**2006**												
18.5–24	5001 (56.5)		1.000 (ref.)		2372 (57.1)		1.000 (ref.)		2629 (55.9)		1.000 (ref.)	
< 18.5	542 (6.1)	− 0.509	0.601 (0.460,0.786)	< 0.001	246 (5.9)	− 0.292	0.746 (0.523,1.065)	0.107	296 (6.3)	− 0.743	0.476 (0.316,0.716)	< 0.001
≥ 24	3311 (37.4)	0.902	2.465 (2.207,2.753)	< 0.001	1537 (37.0)	0.836	2.308 (1.978,2.693)	< 0.001	1774 (37.8)	0.946	2.576 (2.196,3.021)	< 0.001
**2009**												
18.5–24	4495 (53.8)		1.000 (ref.)		2111 (53.5)		1.000 (ref.)		2384 (54.0)		1.000 (ref.)	
< 18.5	556 (6.7)	− 0.697	0.498 (0.383,0.648)	< 0.001	252 (6.4)	− 0.774	0.461 (0.317,.670)	< 0.001	304 (6.9)	− 0.630	0.533 (0.368,0.771)	0.001
≥ 24	3307 (39.6)	0.860	2.363 (2.122,2.631)	< 0.001	1583 (40.1)	0.799	2.223 (1.912,2.586)	< 0.001	1724 (39.1)	0.899	2.456 (2.107,2.864)	< 0.001
**2011**												
18.5–24	6291 (50.4)		1.000 (ref.)		2893 (49.6)		1.000 (ref.)		3398 (51.0)		1.000 (ref.)	
< 18.5	626 (5.0)	− 1.011	0.3640 (0.276,0.480)	< 0.001	256 (4.4)	− 0.854	0.426 (0.288,0.630)	< 0.001	370 (5.6)	− 1.189	0.305 (0.205,0.452)	< 0.001
≥ 24	5573 (44.6)	1.031	2.803 (2.561,3.068)	< 0.001	2679 (46.0)	1.031	2.804 (2.470,3.183)	< 0.001	2894 (43.4)	0.998	2.713 (2.384,3.087)	< 0.001

Adjusted for age, current smoking and current drinking.

*BMI* Body mass index.

## Discussion

In the present study we determined the secular trends in the PAR of overweight/obesity for hypertension in China between 1991 and 2011. The main findings were as follows. First, the age-standardized prevalence of hypertension in China gradually increased between 1991 and 2011 with an increased age-standardized prevalence of overweight/obesity. Second, although the impact of overweight/obesity on hypertension remained stable, the PAR of hypertension attributed to overweight/obesity increased from 27.1% to 44.6%. The present results suggest that the increase in the prevalence of hypertension in Chinese adults was explained, in part, by the increased prevalence of overweight/obesity.

Several studies regarding secular trends in PAR of overweight/obesity for hypertension are inconsistent with our findings^7–10,17^. Among a Japanese population, the age-standardized prevalence of overweight/obesity increased, while the prevalence of hypertension decreased from 1980 to 2010^7^. The decrease in hypertension may have been caused by a decline in other determinants, such as salt intake; however, the authors also mentioned that even though there was no corresponding increase in the prevalence of hypertension, an increase in hypertension may recur in the future. In addition, one recent study that focused on Chinese children 7–17 years of age showed that the prevalence of overweight children increased from 1995 to 2014, while the prevalence of hypertension remained relatively stable. The PAR of hypertension attributed to overweight steadily increased from 6.3% in 1995 to 19.2% in 2014, mainly because of the strong impact of overweight/obesity on hypertension^17^.

According to the PAR formula, the prevalence and OR are determinants of PAR. Our results demonstrated that the OR (the association between overweight and hypertension), did not change during this period. Thus, the increase in hypertension may be caused to a large extent by the increase in obesity. As the results showed, the overweight/obesity epidemic caused an increase in the prevalence of hypertension and the PAR increased to 44.6%. Moreover, a high PAR suggests that controlling overweight/obesity will greatly reduce the prevalence of hypertension, thereby reducing the burden of hypertension-induced CVD in China.

In addition, although the prevalence of hypertension in Chinese children has not increased significantly during the past two decades, the current prevalence of overweight children in China is already at a high level (18.4% in 2014)^17^. Children who are overweight are at increased risk of being overweight and hypertensive in adulthood, which is a major public health problem that needs to be addressed. Therefore, the relationship between excessive BMI and hypertension increases the urgency in addressing the overweight and obesity epidemic.

Based on the recommendation of the American College of Sports Medicine, moderate intensity exercise can prevent weight gain with appropriate dietary intervention^18^. A study involving 120,877 U.S. adults showed that weight gain is inversely associated with a diet high in fruits, vegetables, whole grains, nuts, and yogurt. Lifestyle factors were independently associated with long-term weight gain, including physical activity, alcohol consumption, cigarette smoking, sleep, and television viewing time^19^. These findings indicated that sufficient physical activity, a reasonable diet, and lifestyle changes can lead to a reduction and control of body weight. National policies and strategies may effectively drive people’s attention to their weight and the associated risk factors, thus controlling BP and reducing the burden of CVD.

### Strength and limitations

The strength of our study was that data were collected from serial surveys over a 20-year period. Height, weight, and BP were measured using standardized methods in each survey. The limitations of our study should also be considered. First, the current study was based on cross-sectional surveys distributed in China. Some overweight or obese participants may have been misclassified as normal weight because they attempted to lose weight, which may underestimate the prevalence of overweight and obesity. Second, other factors that may lead to an increased or decreased risk of hypertension, such as dietary factors, were not taken into consideration because there was no information available. Corollary studies, which control for more confounding factors, are warranted to further confirm our results in the future.

## Conclusion

In the past two decades, the increase in the prevalence of hypertension in Chinese adults was partly due to the overweight/obesity epidemic, which serves as an impetus to control weight. National policies and strategies are urgently needed to prompt people to follow healthy diets, increase exercise, reduce excess weight, and to further control BP and reduce the burden of CVD.

## Data Availability

This study uses data from the China Health and Nutrition Survey (CHNS), which is an open database. The datasets used or analyzed during the current study are available from the URL (https://www.cpc.unc.edu/projects/china/data/questionnaires) or the corresponding author upon reasonable request.

## Abbreviations

BP: Blood pressure
CVD: Cardiovascular disease
CHNS: China Health and Nutrition Survey
CIs: Confidence intervals
DBP: Diastolic BP
ORs: Odds ratios
PAR: Population attributable risk
SBP: Systolic BP
